# *Actinidia chinensis* Planch Ameliorates Photoaging in UVB-Irradiated NIH-3T3 Cells and SKH-1 Hairless Mice by Controlling the Reactive Oxygen Species/AKT Pathway

**DOI:** 10.3390/antiox13091091

**Published:** 2024-09-06

**Authors:** Jong-Min Jung, Seo-Young Kim, Oh-Yun Kwon, Seung-Ho Lee

**Affiliations:** Department of Nano-Bioengineering, Incheon National University, 119 Academy-ro, Incheon 22012, Republic of Korea; jjm9659@gmail.com (J.-M.J.); seozero67@naver.com (S.-Y.K.); ohyun1220@naver.com (O.-Y.K.)

**Keywords:** antiphotoaging, epidermal fat, procollagen type 1, metalloproteinase, wrinkle formation

## Abstract

In this study, we evaluated the antiphotoaging properties of *Actinidia chinensis* Planch (ACP) and the molecular mechanisms underlying its ability to prevent UVB-mediated photoaging. Administration of the ethanolic extract of ACP (EEACP) to the dorsal area of hairless mice effectively ameliorated UVB-mediated wrinkle formation, epidermal thickening, and loss of lipid droplets in the epidermis. Additionally, the UVB-induced loss of collagen content in the epidermis was significantly attenuated in mouse skin treated with EEACP. The expression of procollagen type 1 and metalloproteinase-1a, which are related to collagen content in the epidermis, was restored by EEACP treatment in UVB-irradiated mice and NIH-3T3 mouse skin fibroblast cells. Interestingly, EEACP effectively ameliorated UVB-induced reactive oxygen species overproduction. Furthermore, the activation/phosphorylation of AKT, rather than mitogen-activated protein kinases, has been identified as a major target of EEACP in preventing UVB-mediated photoaging. Additionally, N-(1 deoxy-1-fructosyl) valine and phenethylamine glucuronide were identified as analytical indicators of EEACP using high-performance liquid chromatography/mass spectrometry. These results suggest that EEACP can be developed as a functional natural agent capable of preventing photoaging by attenuating UVB-induced activation of the reactive oxygen species/AKT pathway.

## 1. Introduction

Repetitive exposure to ultraviolet (UV) rays is considered a major cause of photoaging and leads to dyspigmentation, wrinkle formation, and subcutaneous (SC) fat loss [1,2,3]. Among the various ranges of UV rays, UVB (280–315 nm), a high-wavelength UV radiation, is regarded as particularly harmful, predominantly inducing detrimental effects on the skin [4]. Chronic exposure to UVB impedes collagen synthesis and accelerates collagen degradation, which is closely associated with the formation of deep wrinkles [5,6]. Consequently, cellular pathways related to collagen biosynthesis in the skin have become targets for the development of antiphotoaging agents.

Excessive UVB irradiation can elicit inflammatory responses in the skin by upregulating the expression of proinflammatory cytokines, such as interleukin (IL)-6, IL-8, and monocyte chemotactic protein-3 (MCP-3) [7]. These proinflammatory cytokines also act as critical mediators of UVB-mediated loss of SC fat [3], and attenuating their abnormal expression in UVB-irradiated skin could effectively prevent photoaging.

Chronic UVB exposure leads to the overproduction of reactive oxygen species (ROS) in skin, which can activate intracellular signaling pathways, such as mitogen-activated protein kinases (MAPKs) and AKT [8]. Numerous studies have suggested that UVB-induced ROS are key regulators of photoaging processes, as ROS-mediated activation of intracellular MAPKs and AKT can trigger the abnormal expression of collagen synthesis-related genes, such as procollagen type 1 (*COL1A1*) and metalloproteinase-1a (*MMP-1a*) [4,9,10]. Several agents that inhibit UVB-induced ROS production have been shown to restore the epidermal collagen content in UVB-irradiated skin [6,11]. Therefore, natural agents that effectively prevent an increase in intracellular ROS in UVB-irradiated skin could serve as potent antiphotoaging agents.

*Actinidia chinensis* Planch (ACP), which belongs to the family Actinidiaceae, is a perennial plant that is widely distributed in East Asia [12]. The fruit of ACP, commonly known as kiwi, has been used as a food additive and in Oriental medicine because of its beneficial effects on dyspepsia, vomiting, and loss of appetite [12]. Numerous studies have demonstrated the promising biological properties of the radix or seed part of ACP, including its effects on tumor progression [13], inflammation [14], bacterial growth [15], and immunoregulation [16]. However, the effects of the fruit part of ACP (Actinidiae Fructus) on UVB-induced skin photoaging have not been elucidated. Therefore, in this study, we aimed to evaluate the effects of ethanol extracts of the fruit part of ACP (EEACP) on UVB-induced dermatological changes and to elucidate the molecular mechanisms by which EEACP regulates photoaging processes. Furthermore, this study suggests single constituents of EEACP that could serve as analytical indicators of EEACP. 

## 2. Materials and Methods

### 2.1. Materials

N-acetyl-L-cysteine (NAC) (A9165) was purchased from Sigma-Aldrich (St. Louis, MO, USA). Rabbit anti-phospho-JNK (9251S), rabbit anti-JNK (9252S), rabbit anti-AKT (9272S), rabbit anti-phospho-AKT (4060S), rabbit anti-ERK1/2 (9101S), rabbit anti-phospho-ERK1/2 (9102S), rabbit anti-phospho-p38 MAPK (9212S), and rabbit anti-p38 MAPK (9211S) antibodies were obtained from Cell Signaling Technology, Inc. (Danvers, MA, USA). Rabbit anti-COL1A1 (PA5-29569) and rabbit anti-MMP-1a (PA5-27210) antibodies were purchased from Invitrogen (Carlsbad, CA, USA). 2′-7′-dichlorofluorescin (DCFH-DA) and dimethyl sulfoxide (DMSO) were obtained from Sigma-Aldrich Co. (St. Louis, MO, USA).

### 2.2. Preparation of EEACP

EEACP was purchased from the Korea Plant Extract Bank (Daejeon, Republic of Korea). ACP (10 g) was ground and extracted with 1 L of ethyl alcohol (95%, *v*/*v*) using an ultrasonic extractor (SDN-900H; SD-Ultrasonic Co., Ltd., Seoul, Republic of Korea). The extracts were filtered and dried under reduced pressure (CleanVac 8; Hanil Science Inc., Gimpo, Republic of Korea). Finally, 0.67 g was obtained. EEACP powder was initially dissolved in a DMSO solution (10%, *v*/*v*) and then diluted (1:1000 or 1:10,000) with phosphate-buffered saline (PBS). The final concentration of DMSO in treatments was kept below 0.01% (*v*/*v*) and the control treatment contained an equal amount of DMSO. Voucher EEACP specimens were deposited at Korea Plant Extract Bank (reference number: PBC-126AS).

### 2.3. Cytotoxicity Assay of EEACP

NIH-3T3 skin fibroblast cells, obtained from the Korean Type Culture Collection (Seoul, Republic of Korea), were maintained in Dulbecco’s modified Eagle’s medium (DMEM) (HyClone, Logan, UT, USA) containing 10% (*w*/*v*) fetal bovine serum (Corning Company, Corning, NY, USA), 100 units/mL penicillin, and 100 μg/mL streptomycin. NIH-3T3 cells (1 × 10^4^/well) were seeded in a 96-well flat-bottomed plate and cultured for 24 h at 37 °C in a CO_2_ incubator. After replacing the culture medium with serum-free DMEM containing EEACP (0–1000 μg/mL), the cells were further incubated for 24 h. The cytotoxicity of EEACP was measured using a cell viability assay kit (Dozen, Seoul, Republic of Korea) according to the manufacturer’s protocol.

### 2.4. Irradiation with UVB

EEACP (10 or 100 µg/mL) was added to the cell culture media and preincubated for 24 h at 37 °C in a CO_2_ incubator. The culture medium was replaced with phosphate-buffered saline (PBS) (1 mL) and irradiated with UVB (25 mJ/cm^2^) using a UV irradiation system (Bio-Link 312; Vilber Co., Suebia, Germany). After UVB irradiation, the cells were further cultured in complete medium (DMEM containing 10% fetal bovine serum, 100 units/mL penicillin, and 100 μg/mL streptomycin) for an additional 24 h. The cells were used to evaluate the expression of various genes.

### 2.5. ROS Measurement

The ROS levels were measured according to the method described by Kwon et al. [17]. Briefly, NIH-3T3 cells (2 × 10^5^/well) were seeded in a 6-well plate (SPL Life Sciences, Gyeonggi, Republic of Korea) and cultured for 24 h. After preincubation with EEACP (10 and 100 µg/mL) or NAC (1 mM) for 24 h at 37 °C in a CO_2_ incubator, the cells were irradiated with UVB (25 mJ/cm^2^) under the condition described above. The cells were washed twice with PBS, and 30 µM of DCFH-DA solution was added to each well. The cells were further incubated for 24 h at 37 °C in a CO_2_ incubator. An equal number of NIH-3T3 cells (1 × 10^4^/100 µL PBS/well) were then transferred to 96-well black plate (SPL Life Sciences, Gyeonggi, Republic of Korea). After further incubation for one hour at 37 °C in a CO_2_ incubator, the intensity of fluorescence was evaluated using a microplate fluorometer (Tecan, Männedorf, Switzerland). NAC (1M, *w*/*v*) was initially dissolved in distilled water and adjusted to pH 8 using a 5N NaOH solution. DCFH-DA was initially dissolved in DMSO solution (10%, *w*/*v*) and diluted (1:1000) with the culture medium (DMEM containing 100 units/mL penicillin, and 100 μg/mL streptomycin). The final concentration of DMSO in treatments was kept below 0.01% (*v*/*v*) and the control treatment contained an equal amount of DMSO.

### 2.6. Animal Experiments

Hairless mice (SKH-1, 6 weeks old) were obtained from Orient Bio, Inc. (Seoul, Republic of Korea). The mice were adapted to their housing conditions for one week under controlled humidity (50 ± 10%), the temperature (23 ± 2 °C) conditions and a 12 h light/dark cycle. The mice were divided into four groups (*n* = 7): control (no treatment), UVB (UVB irradiation and pretreatment with working solution [propylene glycol-ethanol = 7:3]), EEACP 25 + UVB (UVB irradiation and pretreatment with EEACP 25 mg/kg bw), and EEACP 50 + UVB (UVB irradiation and pretreatment with EEACP 50 mg/kg bw). EEACP was dissolved in the working solution and applied to the dorsal area of the mouse skin. The dorsal areas of the mice were initially irradiated with UVB at 50 mJ/cm², with the intensity gradually increased up to 200 mJ/cm². UVB irradiation was performed using a UV irradiation system (Bio-Link 312; Vilber Co., Suebia, Germany) every other day for 10 weeks and the total amount of UVB irradiation was 78 MED. MED (50 mJ/cm^2^) was considered as the dose of UVB radiation required to produce minimal erythema after 24 h.

### 2.7. Skin Replica Assay and Tissue Staining

At the end of the experiment, to assess UVB-induced wrinkle formation, replicas were taken from the dorsal skin of each mouse using silicon impression material (Perfect-F Light Body Cartridge, Handae Chemical, Sungnam, Republic of Korea). Dorsal skin tissues were collected and fixed in a 10% (*w*/*v*) paraformaldehyde solution. Subsequently, the skin tissues were embedded in paraffin, and sections 5 μm thick sections were prepared. These sections were then stained with hematoxylin and eosin to evaluate the changes in epidermal thickness, and with Masson’s trichrome solution to assess the collagen fiber content.

### 2.8. Quantitative Real-Time Polymerase Chain Reaction (qRT-PCR)

To determine the relative expression of photoaging-related genes, transcriptional analysis was conducted using qRT-PCR following the established protocols [18]. Briefly, total RNA was isolated from NIH-3T3 cells and murine skin using TRIzol^®^ reagent (Invitrogen, Waltham, MA, USA). Oligo dT primer (10 µM) and 1 µg of total RNA were used for cDNA synthesis. SYBR^®^ Green Realtime PCR Master Mix (Toyobo Co., Tokyo, Japan) and a RT-PCR detection system (CFX Connect^TM^; Bio-Rad Co., Hercules, CA, USA) were employed for qRT-PCR analysis. The relative expression level of each gene was calculated using the Ct method and normalized to glyceraldehyde 3-phosphate dehydrogenase expression. The primer sequences used in this study are listed in Table 1.

### 2.9. Western Blotting

To evaluate the effects of EEACP on the intracellular signaling pathways activated by UVB irradiation, the phosphorylation status of AKT and MAPK in NIH-3T3 cells was assessed using Western blotting, following a previously described protocol [19]. Briefly, after cell lysis in lysis buffer [20] for 1 h, the cell lysates were centrifuged at 13,000× *g* at 4 °C for 15 min. The protein concentration in the supernatants were determined using the Bradford assay [21]. Subsequently, 20 µg of protein from each sample was separated by sodium dodecyl sulfate polyacrylamide gel electrophoresis and transferred onto nitrocellulose membranes. After blocking with 5% nonfat milk in Tris-buffered saline with Tween 20 for 1 h, the membranes were incubated overnight at 4 °C with primary antibodies (diluted 1:5000). After three washes with Tris-buffered saline with Tween 20, the membranes were incubated with secondary antibodies (diluted 1:3000) conjugated to horseradish peroxidase (Santa Cruz Biotechnology, Dallas, TX, USA) at room temperature (21 °C–23 °C) for 2 h. The protein bands were visualized using an enhanced chemiluminescence detection kit (Bio-Rad, Hercules, CA, USA).

### 2.10. Single Components Analysis

The single components in EEACP were identified using an AQUITY Ultra Performance LC™ system (Waters Corp., San Jose, CA, USA) coupled with a Micromass Q-Tof Premier™ mass spectrometer (Waters Corp., San Jose, CA, USA). EEACP (1 mg/mL) was dissolved with eluent A (aqueous formic acid solution, 0.1% *v*/*v*) and 2 uL of the EEACP solution was injected for analysis. The constituents of EEACP were separated in a BEH C18 column (1.7 µm particle size, 100 mm × 2.1 mm) (Thermo Fisher Scientific, San Jose, CA, USA) using a mobile phase consisting of eluent A and eluent B (acetonitrile with formic acid, 0.1% *v*/*v*). The flow rate was set at 0.4 mL/min under a gradient elution program (0–1 min 100% A; 1–12 min 100% B; 12–13.40 min 100% B; 13.40–13.50 min 100% A; 13.50–15.00 min 100% A) The column temperature was maintained at 40 °C. The Micromass Q-Tof Premier™ mass spectrometer was operated in negative ionization mode with a mass range from 100 to 2000 Da, a scan time of 0.2 s, and a desolvation temperature of 350 °C. The desolvation gas flow rate was 800 L/h (N_2_), the source temperature was 110 °C, the cone voltage was 50 V, and the capillary voltage was 2.3 kV.

### 2.11. Statistical Analysis

All data are presented as mean ± standard deviation. Statistical analyses were performed using Prism version 5 (GraphPad Software, Inc., San Diego, CA, USA). A two-tailed, unpaired Student’s t-test was used for comparisons between two groups, whereas analysis of variance, followed by post hoc tests, was used for comparisons between multiple groups. A *p*-value of less than 0.05 (*p* < 0.05) was considered statistically significant.

## 3. Results

### 3.1. Administration of EEACP Attenuated UVB-Induced Wrinkle Formation

**Figure 1:**
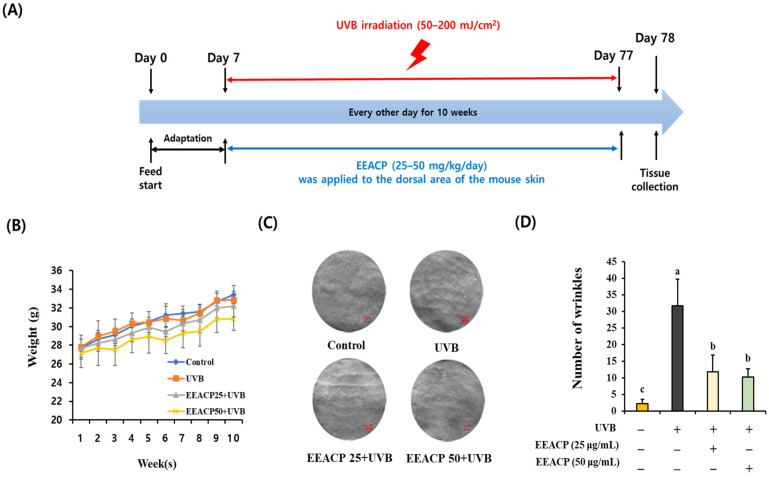
Administration of the ethanolic extract of *Actinidia chinensis* Planch (EEACP) attenuated UVB-induced wrinkle formation. Each mouse group was irradiated with UVB for 10 weeks, with or without EEACP treatments (25–50 mg/kg/day) (**A**). Body weight of each mouse group was measured over 10 weeks (**B**). Wrinkle formation in each mouse group (*n* = 7) was assessed by replica assay (scale bar =1 µm) (**C**) and counted under a microscope (**D**). Data are expressed as mean ± standard deviation (SD). Different letters (a, b, and c) indicate significant differences between groups (*p* < 0.05).

To assess the impact of EEACP on UVB-induced wrinkle formation, we initially examined the changes in body weight among the mouse groups during UVB irradiation and found no significant differences (*p* > 0.05) (Figure 1A). However, significant differences were observed in the number of wrinkles on the dorsal skin between the groups (Figure 1B,C and Figure S1). Specifically, the number of wrinkles significantly increased in the UVB-exposed group (31.66 ± 7.99) compared with that in the control group (2.16 ± 1.34) (*p* < 0.05). Importantly, EEACP treatment significantly reduced the formation of wrinkles (EEACP25:11.83 ± 4.94 and EEACP50:10.16 ± 2.54) compared with that in the UVB group (*p* < 0.05). These findings indicate that EEACP effectively prevented UVB-induced wrinkle formation.

### 3.2. UVB-Induced Epidermal Thickening Was Restored by EEACP Treatments

**Figure 2:**
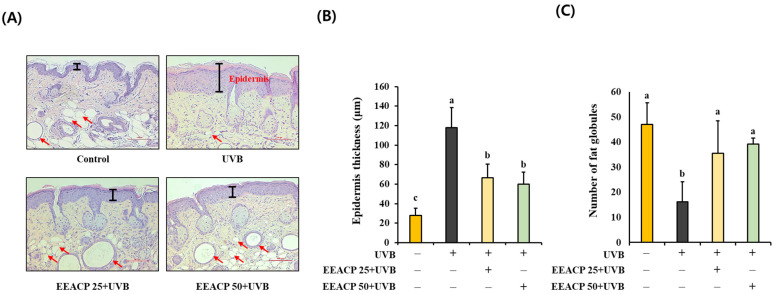
Ethanolic extract of *Actinidia chinensis* Planch (EEACP) ameliorated UVB-induced epidermal thickening and loss of epidermal fat. Skin tissues of each mouse group were isolated and visualized with hematoxylin and eosin (H&E) staining (scale bar =100 μm) (**A**). Epidermal thickness was quantified using Image J software (version 1.8.0) (**B**). The number of epidermal fat globules was counted under a microscope (**C**). Data are expressed as mean ± standard deviation (SD). Different letters (a, b, and c) indicate significant differences between groups (*p* < 0.05). Red arrow indicates fat globules.

Following the evaluation of wrinkle formation, we investigated the epidermal changes in each mouse group. UVB irradiation led to a significant increase in epidermal thickness (117.75 ± 20.87) compared with that in the control group (Figure 2A,B). However, this thickening was notably reduced in mice treated with EEACP (EEACP25:66.45 ± 14.18 and EEACP50:60.12 ± 12.24) (*p* < 0.05). Additionally, the number of fat globules in the epidermis was significantly decreased in the UVB group compared with that in the control group; however, EEACP treatment restored fat globule levels in the skin (Figure 2A,C). These results suggest that EEACP effectively mitigated UVB-induced epidermal thickening and prevented the loss of fat globules in UVB-irradiated mouse skin. Collectively, these findings demonstrate the potential of EEACP as a protective agent against UVB-induced skin damage, specifically targeting wrinkle formation and epidermal changes associated with photoaging. 

### 3.3. The Loss of Collagen Contents in UVB-Irradiated Skin Was Resolved by EEACP Treatments

**Figure 3:**
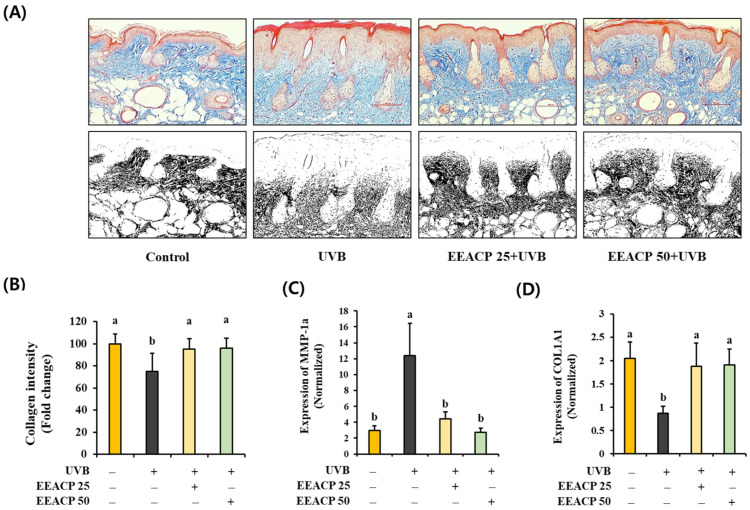
Ethanolic extract of *Actinidia chinensis* Planch (EEACP) attenuated UVB-induced reduction of epidermal collagen. Skin tissues of each mouse group were stained with Masson’s trichrome solution (scale bar =100 μm) (**A**) and analyzed for collagen density using Image J software(version 1.8.0) (**B**). Expression of metalloproteinase-1a (*MMP−1a*) (**C**) and procollagen type 1 (*COL1A1*) (**D**) genes in skin tissues was quantified by quantitative real-time PCR (qRT-PCR). Data are expressed as mean ± standard deviation (SD). Different letters (a, b) indicate significant differences between groups (*p* < 0.05).

During photoaging, UVB exposure can reduce epidermal collagen content. Masson’s trichrome staining was performed to evaluate the effects of EEACP on UVB-induced collagen loss (Figure 3A). The intensity of collagen content significantly decreased in the UVB-exposed group (75.02 ± 16.37) compared with that in the control group (100 ± 8.70). However, treatment with EEACP (EEACP25:95.25 ± 9.14 and EEACP50:95.86 ± 9.00) significantly attenuated this loss significantly (Figure 3B).

Furthermore, EEACP treatment inhibited the UVB-induced expression of *MMP-1a*, a critical enzyme involved in collagen degradation, and enhanced the expression of *COL1A1*, which is crucial for collagen synthesis (Figure 3C,D). These findings suggest that EEACP effectively mitigates UVB-induced loss of epidermal collagen by regulating the expression of collagen-related genes.

### 3.4. EEACP Attenuated the Abnormal Expression of Photoaging-Related Genes in UVB-Irradiated NIH-3T3 Skin Fibroblast Cells

**Figure 4:**
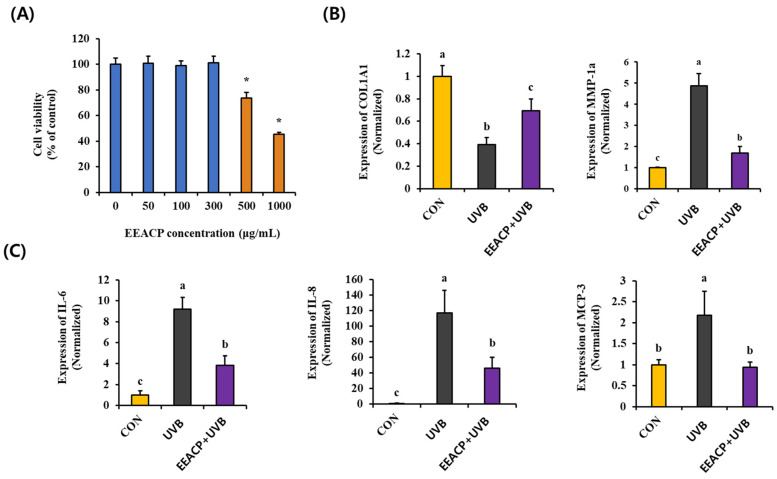
Non-toxicological levels of ethanolic extract of *Actinidia chinensis* Planch (EEACP) showed antiphotoaging properties in NIH-3T3 cells. Cytotoxicity of various concentrations of EEACP (0–1000 µg/mL) was assessed in NIH-3T3 cells (**A**), and non-toxicological levels (100 µg/mL) inhibited UVB-induced expression of procollagen type 1 (*COL1A1*) and metalloproteinase-1a (*MMP-1a*) (**B**). UVB-mediated upregulation of *interleukin (IL)-6*, *IL-8*, and *MCP-3* was attenuated by EEACP (100 µg/mL) (**C**). Gene expression was measured by quantitative real-time PCR (qRT-PCR). Data are expressed as mean ± standard deviation (SD). Different letters (a, b, and c) indicate significant differences between groups (*p* < 0.05). *: Indicates a significant difference (*p* < 0.05) compared with the control (non-treated).

To further investigate the antiphotoaging effects of EEACP, we examined its effect on the expression of photoaging-related genes in UVB-irradiated NIH-3T3 cells. Initially, we determined the nontoxic concentrations of EEACP (up to 100 µg/mL) using a cell viability assay (Figure 4A). Subsequently, we observed that UVB-induced abnormal expression of the *COL1A1* and *MMP-1a* genes was significantly normalized by EEACP treatment at nontoxic concentrations (100 µg/mL) in NIH-3T3 cells (Figure 4B).

Additionally, EEACP treatment reduced the UVB-induced upregulation of proinflammatory cytokines, such as *IL-6*, *IL-8*, and *MCP-3*, which are implicated in the UVB-induced loss of epidermal fat (Figure 4C). These results underscore the role of EEACP in regulating the UVB-mediated abnormal expression of photoaging-related genes.

### 3.5. UVB-Induced ROS Production Was Attenuated by EEACP

**Figure 5:**
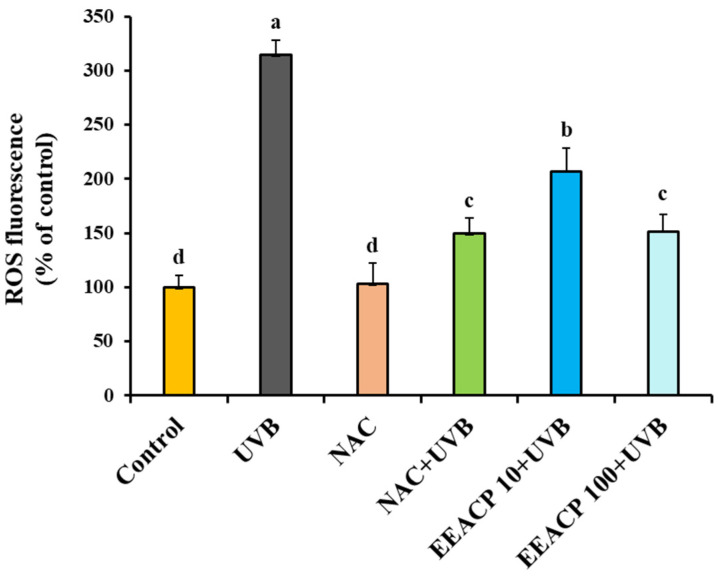
Ethanolic extract of *Actinidia chinensis* Planch (EEACP) restored UVB-mediated dysregulation of reactive oxygen species (ROS) in NIH-3T3 cells. NIH-3T3 cells were pretreated with EEACP (10 and 100 µg/mL) or NAC (1 mM) before UVB irradiation. ROS production was measured using a microplate fluorometer. Experiments were repeated at least three times with similar results. Data are expressed as means ± standard deviation (SD). Different letters (a, b, c, and d) indicate significant differences between groups (*p* < 0.05).

Excessive ROS production is a hallmark of UVB-induced skin damage. Therefore, we evaluated the effect of EEACP on ROS production in UVB-irradiated NIH-3T3 cells. As shown in Figure 5, ROS production significantly increased in UVB-irradiated NIH-3T3 cells (314.70 ± 13.38) compared with that in the control (100.00 ± 10.54). Treatment with nontoxic concentrations of EEACP (EEACP10:207.21 ± 21.49 and EEACP100:151.16 ± 15.42) significantly attenuated ROS production (Figure 5).

Interestingly, the treatments with EEACP (100 µg/mL) exhibited efficacy comparable with that of NAC (1 mM), a potent ROS inhibitor, in reducing UVB-induced ROS production (Figure 5). These findings highlighted EEACP as a potential agent for attenuating UVB-induced intracellular ROS production in NIH-3T3 cells.

### 3.6. EEACP Prevented UVB-Induced Intracellular ROS-Mediated Signaling

**Figure 6:**
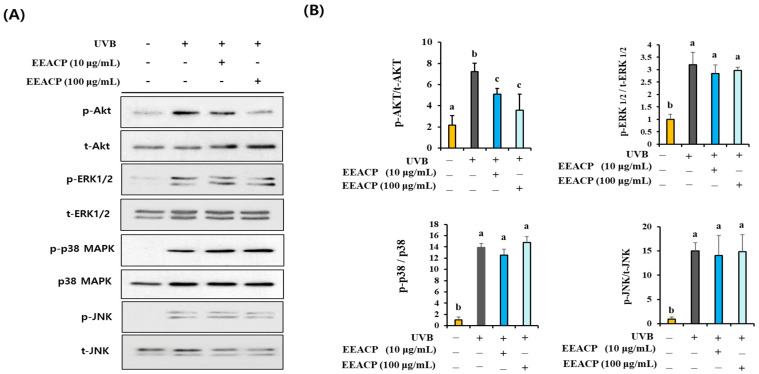
Effects of ethanolic extract of *Actinidia chinensis* Planch (EEACP) on AKT and mitogen activated protein kinases (MAPKs) were assessed by Western blotting (**A**). Band densities were quantified using Image J software (version 1.8.0), and activation ratios of p-AKT/t-AKT and p-MAPKs/t-MAPKs were tabulated (**B**). Experiments were repeated at least three times with similar results. Data are expressed as mean ± standard deviation (SD). Different letters (a, b, and c) indicate significant differences between groups (*p* < 0.05).

To elucidate the intracellular signaling pathways involved in the EEACP-mediated antiphotoaging effects, we assessed the effects of EEACP on the UVB-induced activation and phosphorylation of MAPKs and AKT in NIH-3T3 cells (Figure 6A). Notably, EEACP did not restore the UVB-induced activation and phosphorylation of MAPKs (ERK 1/2, p38 MAPK, and JNK). However, EEACP effectively (*p* < 0.05) attenuated the UVB-induced overactivation/phosphorylation of AKT (Figure 6A,B), suggesting that the AKT signaling pathway may play a central role in the antiphotoaging effects of EEACP.

### 3.7. EEACP and ROS Inhibitor Showed Equivalent Efficacy in Preventing Photoaging

**Figure 7:**
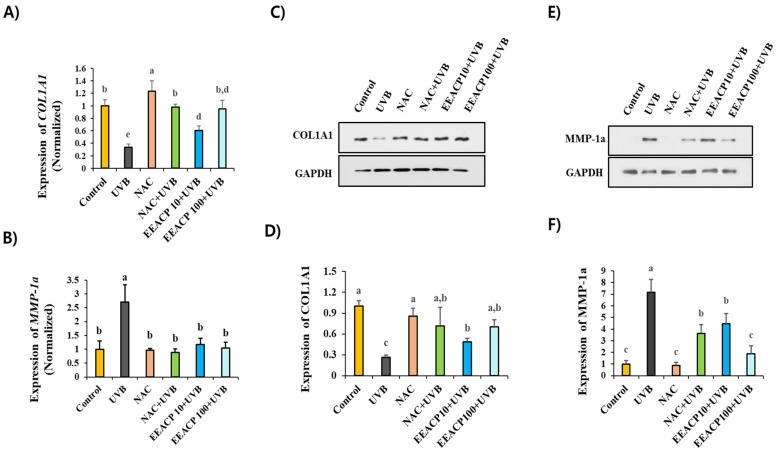
Ethanolic extract of *Actinidia chinensis* Planch (EEACP) exhibited equivalent antiphotoaging activity to a reactive oxygen species (ROS) inhibitor. Antiphotoaging effects of EEACP (10 and 100 µg/mL) were compared with those of NAC (1 mM) on UVB-induced expression of procollagen type 1 (*COL1A1*) and metalloproteinase-1a (*MMP-1a*). NIH-3T3 cells were pretreated before UVB irradiation, and gene expression was assessed by quantitative real-time PCR (qRT-PCR) for *COL1A1* (**A**) and *MMP-1a* (**B**). Protein levels were evaluated by Western blotting for COL1A1 (**C**) and MMP-1a (**E**). Band densities were quantified using Image J software (version 1.8.0) and tabulated (**D,F**). Experiments were repeated at least three times with similar results. Data are expressed as mean ± standard deviation (SD). Different letters (a, b, and c) indicate significant differences between groups (*p* < 0.05).

To assess the efficacy of EEACP in preventing photoaging, we compared its effects with those of NAC, a ROS inhibitor, on *COL1A1* and *MMP-1a* expression in UVB-irradiated NIH-3T3 cells. As shown in Figure 7, EEACP (100 µg/mL) demonstrated equivalent efficacy to NAC (1 mM) in restoring the abnormal expression of *COL1A1* and *MMP-1a* genes (Figure 7A,B). Additionally, EEACP effectively restored the protein levels of COL1A1 and MMP-1a in UVB-irradiated NIH-3T3 cells with an efficacy comparable to that of NAC (Figure 7C–F). These results indicate that ROS are the major targets of EEACP in regulating the photoaging process.

### 3.8. Single Constituents of EEACP Identified by High-Performance Liquid Chromatography/Mass Spectrometry (HPLC/MS) Analysis

**Figure 8:**
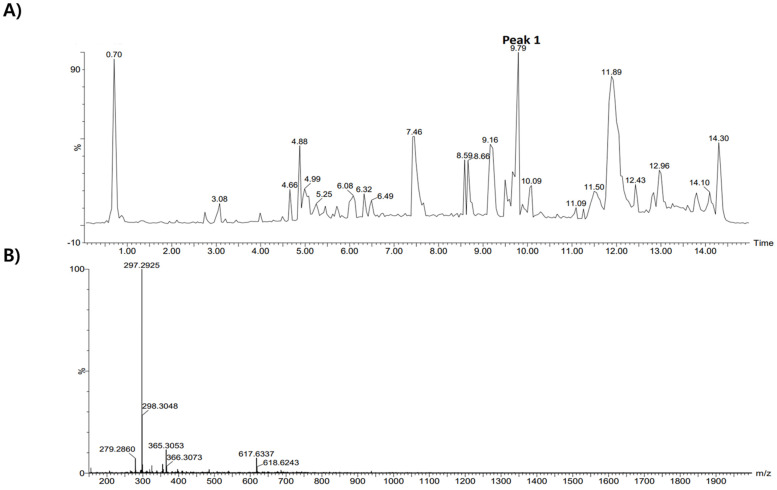
High-performance liquid chromatography/mass spectrometry **(**HPLC/MS) analysis of ethanolic extract of *Actinidia chinensis* Planch (EEACP). Components of EEACP were separated by HPLC (**A**), and a major peak (peak 1) detected at tR 9.79 was further analyzed by mass spectrometry (**B**).

In order to identify the single constituents of EEACP, an HPLC/MS analysis was conducted in negative mode. Several peaks were detected in the HPLC chromatogram of EEACP (Figure 8A). The major peak (tR = 9.79) was subjected to mass analysis (Figure 8B). The observed masses of the major peaks were identified as N-(1-deoxy-1-fructosyl) valine and phenethylamine glucuronide, which served as analytical indicators of EEACP (Table 2). Although their specific antiphotoaging properties were not assessed in this study, these constituents are valuable for the characterization of EEACP.

## 4. Discussion

The skin plays a critical role in maintaining the balance of body fluids and regulating body temperature [24]. Its primary function is to protect against external risk factors, such as UV radiation, viruses, bacteria, and pollutants. It is well-known that if the skin barrier is compromised, various physiological abnormalities and metabolic disorders may occur in the body [25,26]. Among environmental risk factors, UVB radiation has garnered significant attention owing to its ability to cause deep wrinkles and damage the skin barrier through repetitive exposure. Therefore, it is crucial to develop safe and effective anti-UVB agents to prevent UVB-induced photoaging.

In this study, we evaluated the antiphotoaging efficacy of EEACP. The administration of nontoxic levels of EEACP effectively mitigated wrinkle formation, epidermal thickening, and loss of epidermal fat. Furthermore, EEACP treatment attenuated the reduction in epidermal collagen content in UVB-irradiated mouse skin. Collectively, these findings strongly suggest that EEACP has the potential to prevent UVB-induced photoaging.

The reduction in SC fat in UVB-irradiated skin is considered a major aspect of photoaging. As UVB cannot penetrate the skin barrier to reach the epidermal fat, mediators that play critical roles in the UVB-mediated loss of SC fat have been implicated. Interestingly, the induction of proinflammatory cytokines, such as IL-6, IL-8, and MCP-3, has been proposed as a functional mediator that stimulates SC fat loss in UVB-irradiated skin. For example, several natural agents, including *Nelumbo nucifera* leaf extracts, kaempferide, and water extracts of *Alpinia officinarum* rhizome, have demonstrated inhibitory properties against the UVB-induced reduction in SC fat and increases in the expression of IL-6, IL-8, and MCP-3 [11,27,28]. Additionally, Kim et al. showed that blocking the expressions of these cytokines with specific antibodies restored the downregulation of lipid synthesis-related genes in UVB-irradiated skin fibroblasts [29]. Our study found that EEACP treatment significantly restored the UVB-mediated reduction in SC fat and increased IL-6, IL-8, and MCP-3 levels. Collectively, our results suggest that EEACP ameliorates the UVB-induced reduction in SC fat by regulating the expression of SC fat mediators, such as IL-6, IL-8, and MCP-3.

Abnormal expression of photoaging-related genes is often detected in UVB-irradiated skin owing to the activation of intracellular signaling pathways involved in the transcriptional activation of these genes by UVB. ROS-triggered intracellular signaling pathways (ROS/MAPKs or ROS/AKT) are well-known pathways that control the UVB-mediated activation of photoaging-related genes, such as *COL1A1* and *MMPs* [30]. In our previous study, we observed that increased intracellular ROS levels are closely associated with the activation of MAPKs and AKT in UVB-irradiated skin fibroblasts [11]. Moreover, the restoration of transcriptional abnormalities in UVB-irradiated skin fibroblasts by treatment with ROS inhibitors indicated that ROS-mediated signaling pathways could be targeted to inhibit the photoaging process. In this study, we found that EEACP administration attenuated the UVB-induced production of intracellular ROS. Additionally, EEACP was found to inhibit AKT activation but not MAPK activation, suggesting that the antiphotoaging properties of EEACP may be mediated through the specific regulation of the ROS/AKT pathway.

ACP is a natural agent used in traditional medicine. While several studies have demonstrated the biological activities of ACP, our study is the first, to our knowledge, to show the antiphotoaging activity of ACP and its underlying mechanisms. Based on our results, identifying the functional single constituent of EEACP could reveal it as a promising natural candidate for an antiphotoaging agent. Several studies have examined the single components of the radix or seed parts of ACP [14,31,32], but chemical analysis of the fruit part is rarely conducted. Therefore, we used HPLC/MASS analysis to identify the components of EEACP, which led to the identification of N-(1-Deoxy-1-fructosyl) valine and phenethylamine glucuronide as major constituents. We found that neither N-(1-Deoxy-1-fructosyl) valine nor phenethylamine glucuronide is present in other parts of ACP. Interestingly, although few studies have explored the functional properties of phenethylamine glucuronide, its major structure contains an aromatic amine known to have antioxidant properties [33]. In addition, 2,2′-azino-bis(3-ethylbenzothiazoline-6-sulfonic acid (ABTS) radical scavenger activity and the reducing power of N-(1-deoxy-1-fructosyl) valine have been suggested [34]. Therefore, since UVB-induced ROS production was reduced in NIH-3T3 cells treated with EEACP, phenethylamine glucuronide and N-(1-Deoxy-1-fructosyl) valine might play a critical role as functional components of EEACP. 

This study had some analytical limitations. Although putative single constituents such as N-(1-deoxy-1-fructosyl) valine and phenethylamine glucuronide were identified, their presence or concentration in EEACP could not be elucidated due to the difficulty in obtaining purified standards of these substances. Future studies should aim to identify all single constituents of EEACP through HPLC/MASS profiling analysis. Additionally, the specific active constituents responsible for its antiphotoaging activity will be determined using in vitro and in vivo photoaging models.

## 5. Conclusions

In this study, EEACP was shown to have strong antiphotoaging efficacy. Topical administration of EEACP to the dorsal area of mouse skin effectively prevented UVB-induced wrinkle formation, epidermal thickening, and loss of dermal collagen. In addition, the ROS/AKT signaling pathway was identified as a specific pathway involved in EEACP-mediated antiphotoaging effects. Our results suggest that EEACP could be developed as an antiphotoaging agent capable of attenuating UVB-induced activation of the ROS/AKT signaling pathway.

## Figures and Tables

**Table 1:** Primer sequences.

Genes	Sequences		Species
	Forward	Reverse	
*COL1A1*	5′-CACTGCTGTTGGTCCACGT-3′	5′-AAAGCACAGCACTCGCCC-3′	Mouse
*MMP-1a*	5′-ACTTTCCAGCCAGGCCCA-3′	5′-CACTGCTGTTGGTCCACGT-3′	Mouse
*IL-6*	5′-ACAACCACGGCCTTCCCT-3′	5′-AGCCTCCGACTTGTGAA-3′	Mouse
*IL-8*	5′-TGTCCCATGCCACTCAGAGA-3′	5′-AGCAGGTGCTCCGGTTGTAT-3′	Mouse
*MCP-3*	5′-ATAGCCGCTGCTTTCAGCAT-3′	5′-CTTCCCAGGGACACCGACTA-3′	Mouse
*GAPDH*	5′-AAGCTGTGGCGTGATGGC-3′	5′-AAGCTGTGGCGTGATGGC-3′	Mouse

**Table 2:** Major constituent of EEACP.

Natural Product	Peak	RT (min)	Observed Mass	Fragment ions	Single Compound	Molecular Structure	Formula	Molecular Mass (g/mol)	Ref.
*Actinidia chinensis* Planch	Peak 1	9.79	617.6337	365.3053, 297.2925, 279.2860	N-(1-deoxy-1-fructosyl) valine	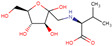	C_11_H_21_NO_7_	279.13	[22]
					Phenethylamine glucuronide	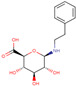	C_14_H_19_NO_6_	297.12	[23]

## Data Availability

The data presented in this study are available upon request from the corresponding author.

## Supplementary Materials

The following supporting information can be downloaded at: https://www.mdpi.com/article/10.3390/antiox13091091/s1.
