# Supplementary material for: Actinidia chinensis Planch Ameliorates Photoaging in UVB-Irradiated NIH-3T3 Cells and SKH-1 Hairless Mice by Controlling the Reactive Oxygen Species/AKT Pathway
Source: Antioxidants (Basel). 2024 Sep 6;13(9):1091. doi: 10.3390/antiox13091091 (PMC11428346; doi:10.3390/antiox13091091)
Supplement: Supplementary file 1 [file antioxidants-13-01091-s001.zip › antioxidants-3157585-supplementary.pdf]

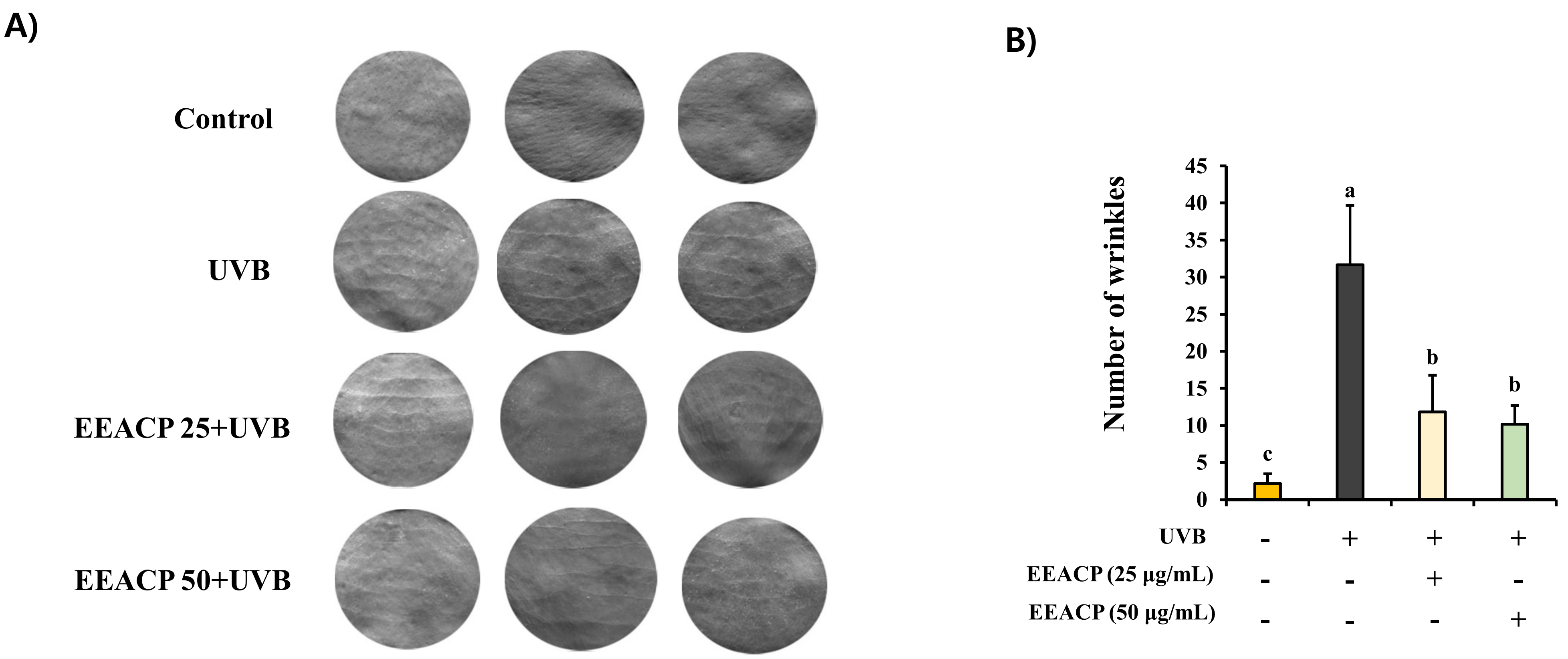

Supplementary Figure S1. Representative images of wrinkle formation in each mouse group (n=7) are showed (A) and the number of wrinkles was counted and tabulated (B). Different letter (a, b, and c) indicate significant differences between groups (p<0.05)
